# Altered rectal sensory response induced by balloon distention in patients with functional abdominal pain syndrome

**DOI:** 10.1186/1751-0759-3-13

**Published:** 2009-11-20

**Authors:** Tsukasa Nozu, Miwako Kudaira

**Affiliations:** 1 Department of Comprehensive Medicine, Hokkaido University Hospital, Kita14, Nishi5, Kita-Ku Sapporo 0608648, Japan

## Abstract

**Background:**

Functional abdominal pain syndrome (FAPS) has chronic unexplained abdominal pain and is similar to the psychiatric diagnosis of somatoform pain disorder. A patient with irritable bowel syndrome (IBS) also has chronic unexplained abdominal pain, and rectal hypersensitivity is observed in a majority of the patients. However, no reports have evaluated the visceral sensory function of FAPS precisely. We aimed to test the hypothesis that FAPS would show altered visceral sensation compared to healthy controls or IBS. The present study determined the rectal perceptual threshold, intensity of sensation using visual analogue scale (VAS), and rectal compliance in response to rectal balloon distention by a barostat in FAPS, IBS, and healthy controls.

**Methods:**

First, the ramp distention of 40 ml/min was induced and the thresholds of discomfort, pain, and maximum tolerance (mmHg) were measured. Next, three phasic distentions (60-sec duration separated by 30-sec intervals) of 10, 15 and 20 mmHg were randomly loaded. The subjects were asked to mark the VAS in reference to subjective intensity of sensation immediately after each distention. A pressure-volume relationship was determined by plotting corresponding pressures and volumes during ramp distention, and the compliance was calculated over the linear part of the curve by calculating from the slope of the curve using simple regression.

**Results:**

Rectal thresholds were significantly reduced in IBS but not in FAPS. The VAS ratings of intensity induced by phasic distention (around the discomfort threshold of the controls) were increased in IBS but significantly decreased in FAPS. Rectal compliance was reduced in IBS but not in FAPS.

**Conclusion:**

An inconsistency of visceral sensitivity between lower and higher pressure distention might be a key feature for understanding the pathogenesis of FAPS.

## Background

Functional gastrointestinal disorders (FGIDs) are characterized as chronic or recurrent gastrointestinal symptoms that are not explained by structural or biochemical abnormalities. They are diagnosed by Rome criteria [1]. Functional abdominal pain syndrome (FAPS), one of the FGIDs, is defined as "pain for at least six months that is poorly related to gut function and associated with some loss of daily activities". The prevalence is reported to be much less than irritable bowel syndrome (IBS) or other FGIDs [2], but this disease has a great impact on quality of life and on the medical economy, because the patients miss many work or school days and have high health care resource use [2].

The pathogenesis of this disease is poorly understood, but abnormality of pain modulation, particularly at the central level, is thought to be the possible cause. However, only a few studies addressing the visceral sensory function of FAPS have been reported so far [3,4], and there is no agreement on whether FAPS patients have altered visceral sensory function. Recently, we demonstrated reduced rectal pain threshold in response to balloon distention in IBS, but no difference between FAPS and healthy controls, suggesting the visceral sensory function of FAPS patients may not be altered, in contrast to IBS [5].

The aim of the present study is to do a detailed assessment of the sensory response induced by rectal distention in FAPS patients. We evaluated rectal perceptual threshold, perceived intensity by visual analogue scale (VAS), and rectal compliance in response to rectal balloon distention by a barostat in FAPS, IBS and healthy controls to clarify the possible pathogenesis of FAPS.

## Methods

### Subjects

Controls. Thirteen healthy subjects (7 women and 6 men) were recruited by advertisement to serve as controls. All had normal bowel habits, and none had known gastrointestinal disease, was taking medication, or had a history of acute or chronic illness.

Patients. Seven patients with IBS (5 women and 2 men) and 6 patients with FAPS (4 women and 2 men) were recruited from the Department of Comprehensive Medicine, Hokkaido University Hospital. Selection criteria included a positive diagnosis by the Rome II criteria [6]. All the IBS patients were diarrhea type and all the FAPS patients had normal bowel habits. No patients had evidence of organic disease by diagnostic studies including abdominal ultrasonography and upper and lower gastrointestinal endoscopy. All the patients with IBS were taking smooth muscle relaxant and calcium polycarbophil, and two were taking anxiolytics and/or tricyclic antidepressants. On the other hand, half of the patients with FAPS were given anxiolytics and/or tricyclic antidepressants, and two were taking selective serotonin reuptake inhibitors.

To assess the symptom severity, the subjects were asked the number of days work or school was missed because of illness in the past three months and the length of hospital stay because of illness. Verbal and written informed consent was obtained from each subject. This study was approved by the Hokkaido University Ethical Committee on Human Studies.

### Psychological status checklist

All subjects completed the hospital anxiety and depression scale (HADS) questionnaire, which assesses current psychological status regarding anxiety and depression [7,8]. HADS scores ≥ 11 were defined as clinically relevant anxiety or depression, and a cutoff of ≥ 8 was defined as borderline.

### Visceral stimulation device

Distention of the rectum was effected by air inflation of a balloon catheter. A computer-driven barostat device (Synectics Visceral Stimulator; Synectics, Stockholm, Sweden) was used for the evaluation of rectal sensation and compliance. It could be programmed to deliver distention according to various protocols by air inflation of the balloon in the rectum, to record pressures and volumes simultaneously (sampling rate 1 per second), and to log sensations by a push-button marker device onto a data file. The balloon catheter consisted of a balloon (a thin-walled polyethylene balloon) attached to a Silastic elastomer tube (external diameter 18F, MAK-LA; Los Angeles, CA, USA) at both proximal and distal ends. The distance between the attachment sites was 9 cm, and distention to a maximal volume of 500 ml resulted in a spherical balloon shape. The open ends of the tube were connected to the inflation channel and pressure sensor port of the barostat device. Before placement in the rectum, the balloon was checked for air leaks by maintaining an intra-balloon pressure of 20 mmHg for 5 min in water.

### Threshold

Rectal perceptual thresholds such as discomfort, pain or maximum tolerance were determined as intra-balloon pressure (mmHg).

### Rectal compliance

The pressure-volume relationship was determined during rectal balloon distention for each subject by plotting corresponding pressures and volumes. As the compliance curve is S-shaped, the compliance was calculated over the linear part of the curve by calculating from the slope of the curve using simple regression.

### Intensity

Subjective intensity of sensation in response to rectal balloon distention was determined by VAS ranging from no sensation (0) to severe (100) arrayed along a 100-mm bar. The subjects marked the VAS in reference to subjective intensity immediately after each distention.

### Experimental protocol

All medications were discontinued 48 h before the procedure. After a 15-h fast, bowel cleansing was performed by warm water enema (250 ml). Subjects were placed in the left lateral decubitus position on the bed, and the balloon, which was lubricated with olive oil, was inserted into the rectum. Then, the subjects lay prone on the bed. The experimental rectal distention protocol started after a 30-min resting period. First, subjects were given ramp distention. The barostat device was programmed to inflate the balloon at an inflation rate of 40 ml/min. During the ramp distention, we determined three sensory thresholds; discomfort, pain and maximum tolerance; and rectal compliance. When the subject felt the distention was intolerable and pressed the pushbutton (i.e. threshold of maximum tolerance was obtained), the balloon was instantaneously deflated and this first session was finished. Ten minutes later, to determine the intensity of sensation using VAS, three phasic distentions of 10, 15 and 20 mmHg for 60 sec separated by 30-sec intervals at a resting pressure of 0 mmHg, were randomly loaded. These distentions consisted of rapidly inflating the balloon at 14 ml/sec until the target pressure was reached, maintaining it for 60 sec, and finally rapidly deflating it at 14 ml/sec. The subjects were asked to mark the VAS in reference to subjective intensity of sensation immediately after each distention.

### Statistical analysis

All data are expressed as means ± SE. To compare the clinical characteristics and rectal compliance of each group, an analysis of variance (ANOVA) or Kruskal-Wallis one-way ANOVA followed by the least significant difference test (LSD) or the Mann-Whitney rank sum test was performed. Chi-square test was used to compare proportional differences (male and female ratio) among groups. For analysis of group differences in perceived intensity (VAS ratings) or thresholds in response to rectal distention, a three-group (FAPS, IBS and control) × three-loaded pressure (10, 15 and 20 mmHg) or threshold (discomfort, pain and maximum tolerance) ANOVA design was followed by LSD. For these analyses, the main effect of grouping indicates an overall difference among groups (FAPS, IBS and control) across three loaded pressures or thresholds. The main effect of loaded pressure or threshold indicates an overall difference among loaded pressures or thresholds across the groups. A group × distention or threshold interaction indicates that the three groups differed in their response. Statistica (StatSoft Inc. Tulsa, Okla., USA) was used for all statistical computations. An α cutoff of P < 0.05 was used throughout the study.

## Results

Table 1 summarizes the clinical characteristics of the subjects. The mean age of the subjects (Kruskal-Wallis one-way ANOVA: χ^2 ^= 3.5, P > 0.05) and the ratio of males to females (Chi-square test, χ^2 ^= 22.6, P > 0.05) were not significantly different among the groups. Stool frequency,the mean number of bowel movements per day in the most recent two weeks, was significantly greater in IBS as compared with control or FAPS (Kruskal-Wallis one-way ANOVA: χ^2 ^= 12.8, P < 0.05, IBS vs. control or FAPS, P < 0.05). The body mass index was not different (ANOVA: F = 2.5, P > 0.05). The number of days missed from work or school because of illness in the most recent three months was significantly greater in FAPS and IBS as compared to the controls (Kruskal-Wallis one-way ANOVA: χ^2 ^= 20.54, P < 0.05, IBS or FAPS vs. control, P < 0.05). On the other hand, the length of hospital stay because of illness was significantly different among the groups by Kruskal-Wallis one-way ANOVA (χ^2 ^= 7.15, P < 0.05), but pairwise comparisons by Mann-Whitney rank sum test did not demonstrate any significant difference between IBS or FAPS and the controls (P > 0.05). The HADS scores were significantly greater in both groups of patients as compared to the controls (Kruskal-Wallis one-way ANOVA: χ^2 ^= 22.6, P < 0.05, for anxiety, χ^2 ^= 16.4, P < 0.05, for depression, IBS or FAPS vs. control, P < 0.05 for both scores). The mean value of FAPS can be considered definite anxiety.

**Table 1:** Clinical characteristics

Clinical parameters	FAPS	IBS	Control
No. of subjects	6	7	13
Mean age (years)	41.2 ± 4.7	26.4 ± 2.3	32.3 ± 3.4
Sex (M/F)	2/4	2/5	6/7
Stool frequency (/day)	0.92 ± 0.1	2.9 ± 0.6*	1.0 ± 0.1
BMI	21 ± 0.7	21 ± 1.0	20 ± 0.7
Days missed from work or school because of illness over past three months	33.8 ± 14.1*	20.4 ± 3.4*	0 ± 0
Length of hospital stay because of illness (days)	21.8 ± 14.2	7.7 ± 5.4	0 ± 0
HADS			
Anxiety	12.5 ± 1.8*	7.4 ± 1.2*	2.2 ± 0.1
Depression	6.5 ± 1.8*	5.9 ± 1.5*	1.9 ± 0.2

FAPS, functional abdominal pain syndrome; IBS, irritable bowel syndrome; BMI, body-mass index; HADS, hospital anxiety and depression scale

*P vs. control <0.05; Kruskal-Wallis one-way analysis of variance followed by the Mann-Whitney rank sum test

**Figure 1:**
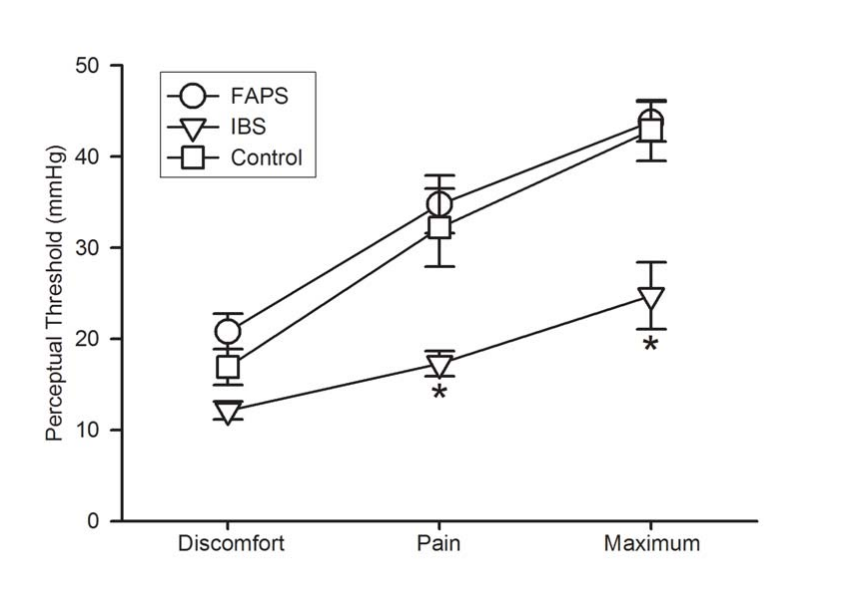
**Thresholds of discomfort, pain and maximum tolerance in response to ramp distention**. Values are shown as mean ± SE. * P vs. control < 0.05, analysis of variance followed by the least significant difference test. FAPS, functional abdominal pain syndrome; IBS, irritable bowel syndrome.

Figure 1 shows the perceptual thresholds in response to rectal distention. The threshold increased in the order of discomfort, pain and maximum tolerance across the groups (ANOVA: F = 75.8; P < 0.05). On the other hand, there was a significant main effect of group (ANOVA: F = 6.6; P < 0.05). IBS had significantly lower threshold as compared to control or FAPS (P < 0.05), and it was not different between FAPS and control (P > 0.05). Discomfort threshold was not different between control and IBS (12.1 ± 1.0 mmHg for IBS vs. 16.9 ± 2.0 mmHg for control, P > 0.05), but pain threshold and maximum tolerance were reduced in IBS (pain threshold; 17.3 ± 1.4 mmHg for IBS vs. 32.2 ± 4.3 mmHg for control, P < 0.05, maximum tolerance; 24.7 ± 3.7 mmHg for IBS vs. 42.9 ± 3.3 mmHg for control, P < 0.05). On the other hand, thresholds were not different between FAPS and control (discomfort threshold; 20.8 ± 2.0 mmHg, pain threshold; 34.8 ± 3.2 mmHg and maximum tolerance; 43.8 ± 2.2 mmHg for FAPS vs. control, P > 0.05). Significant interaction (group × threshold, F = 3.51, P < 0.05) was also observed.

**Figure 2:**
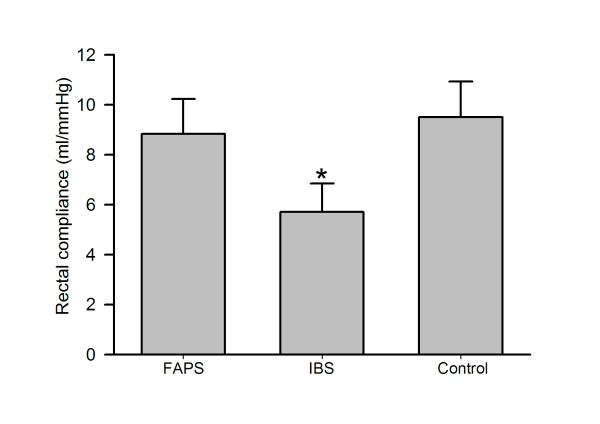
**Rectal compliance in response to ramp distention**. Values are shown as mean ± SE. * P vs. control < 0.05, analysis of variance followed by the least significant difference test.

The rectal compliance was also significantly reduced in IBS (Fig. 2, ANOVA: F = 3.73; P < 0.05, 5.7 ± 1.1 ml/mmHg for IBS vs. 9.8 ± 1.0 ml/mmHg for control, P < 0.05) but it was not different between control and FAPS (9.7 ± 1.2 ml/mmHg for FAPS vs. control, P > 0.05).

**Figure 3:**
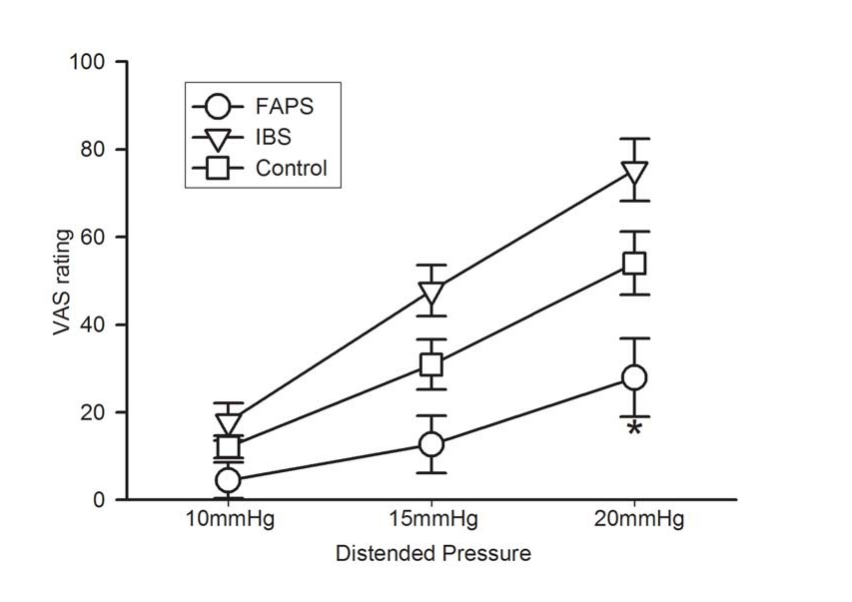
**Intensity of sensation assessed by visual analogue scale (VAS) at three different phasic distentions**. Values are shown as mean ± SE. * P vs. control < 0.05, analysis of variance followed by the least significant difference test.

Figure 3 shows VAS ratings of sensory intensity. There was a significant main effect of loaded pressure (ANOVA: F = 70.25, P < 0.05), i.e. intensity increased as distended pressure increased from 10 to 20 mmHg. Moreover, a significant main effect of group was also observed (ANOVA: F = 7.53, P < 0.05). FAPS had significantly lower VAS ratings as compared to control or IBS (P < 0.05), and IBS had significantly higher VAS ratings as compared to control (P < 0.05). The VAS ratings at 10 and 15 mmHg were not significantly different between FAPS and control (10 mmHg; 4.5 ± 4.1 for FAPS vs. 12.1 ± 2.6 for control, 15 mmHg; 12.7 ± 6.5 for FAPS vs. 30.9 ± 5.7 for control, P > 0.05), but the value was significantly reduced in FAPS at 20 mmHg (27.9 ± 8.9 for FAPS vs. 54.0 ± 7.2 for control, P < 0.05). Although ANOVA demonstrated IBS had significantly higher VAS ratings across the three loaded pressures, each value tended to be higher, but not significantly different from control (17.9 ± 4.3 at 10 mmHg, 47.8 ± 5.8 at 15 mmHg and 75.3 ± 7.1 at 20 mmHg for IBS vs. control, P > 0.05). Moreover, there was a significant interaction (group × loaded pressure, ANOVA: F = 3.53, P < 0.05).

We calculated the sum of VAS ratings of intensity at three distentions and set the cut off value at 60, high efficiency (85%) with very good sensitivity (83%) and specificity (85%), to discriminate FAPS patients from other subjects.

## Discussion

No definitive neurophysiological study in FAPS has been published to date, but neuropathic pain induced by central sensitization is thought to be the most probable pathogenesis [9]. Central sensitization is characterized by a decrease in threshold, an increase in response duration and magnitude to stimuli, and an expansion of the mechanosensitive receptive field of dorsal horn neurons [10]. In the present study, we assessed perceptual threshold and intensity of sensation induced by rectal distention in order to examine whether FAPS patients have altered visceral sensory function, including central sensitization.

Our study revealed that FAPS had a normal perceptual threshold, but it was reduced in IBS. Previous reports by other researchers demonstrated FAPS had normal rectal sensory threshold [4] and rectal hypersensitivity [3,11]. However, these studies were investigated in children, and no data of adult FAPS patients is available except for our previous brief study demonstrating that they had normal rectal thresholds of pain and maximum tolerance [5]. The present study reconfirmed this result clearly, although it should be noted that in the threshold study the discomfort threshold was not statistically different but tended to be higher in FAPS as compared to the controls (Fig. 1). The percent difference from the control value was + 23% at the discomfort threshold and it was + 8 and + 2% at the pain threshold and maximum tolerance, respectively. On the basis of this finding, we tested the perceived intensity at the distended pressure around the discomfort threshold of the control subjects at 10, 15 and 20 mmHg in order to detect possible differences between FAPS and the controls. Our pilot study revealed that the threshold for first sensation in response to rectal ramp distention was 8.9, 12.2 and 14.9 mmHg for IBS, controls and FAPS, respectively, Moreover, some IBS patients did not tolerate the distention over 20 mmHg. In this context, this range of distention was adequate to assess the perceived intensity of all of the subjects tested in the present study.

The most important point of this study is that FAPS patients demonstrated reduced intensity of sensation. On the other hand, IBS patients had significantly higher perceived intensity, which is consistent with previous reports [12,13]. These results suggest that altered visceral sensory function may be observed not only in IBS but also in FAPS. However, the altered pattern was opposite, i.e. hypersensitivity for IBS but hyposensitivity for FAPS. Moreover, this hyposensitivity was only induced by non-painful distentions. It is not clear whether this phenomenon actually occurred associated with the pathogenesis or resulted from response bias. A high level of anxiety may influence sensory parameters [14,15], but both IBS and FAPS patients with high anxiety scores on HADS showed completely different sensory profiles, suggesting anxiety did not modify the results in the present study. Moreover, we could discriminate FAPS patients from other subjects successfully using VAS ratings, suggesting this result may not be induced by bias but that it is related to the pathogenesis to some extent. FAPS overlaps the psychiatric diagnosis of somatoform pain disorder (pain disorder associated with psychological factors) in the DSM-IV, wherein symptoms are localized to the abdomen [16], and any of several psychiatric diagnosis such as depression, anxiety, personality disorders or other somatoform disorders frequently coexist [9]. In this context, this syndrome frequently needs early psychological treatment, and accurate and prompt diagnosis is very important. Because discrimination between IBS and FAPS is sometimes difficult, our procedure would seem to be useful for clinical practice.

The mechanism of this response remains unclear. Rectal compliance was reduced in IBS, which is consistent with the previous reports [17,18]. However, it was not different between FAPS and the controls, suggesting rectal tone does not seem to contribute. The rectum is innervated by both pelvic and lumbar splanchnic nerves. The pelvic nerve afferents are activated at lower stimulation intensities and they mediate non-noxious physiological sensations such as the presence of stool or gas. On the other hand, lumbar splanchnic afferents, with their higher stimulus response threshold, would be better tuned to signal the onset of higher-intensity mechanical events, such as muscular contraction or passage of material [19]. These lines of evidence suggest that hyposensitivity at lower pressure of distention might result from altered pelvic nerve function in FAPS. A number of investigators have reported the presence of rectal hyposensitivity in patients with constipation, which may result from altered pelvic nerve activity [20,21]. This alteration induces abolished perceptual response to physiological stimuli such as rectal filling by stool, which is thought to lead to constipation. Although all the patients with IBS tested in the present study were of the diarrhea type who demonstrated rectal hypersensitivity, IBS with constipation was also reported to include hypersensitivity [22]. However, interestingly, at the same time, hyposensitivity to physiological stimuli was also detected in constipated IBS [22]. These lines of evidence suggest that selectively altered pelvic nerve function is not so unique a phenomenon and it comes as no surprise that this change occurs in FAPS.

We do not know the meaning of this response in the pathogenesis; moreover, it is not clear whether this result is contrary to possible pathogenesis, i.e. central sensitization. Peripheral neuropathic conditions resulting from various types of nerve injury could provide ongoing afferent input to the spinal cord, keeping it in a constant state of central sensitization [23]. Such nerve injury could result from abdominal surgeries or injuries to pelvic nerves during pregnancy or delivery. In fact, some FAPS patients were reported to undergo several abdominal surgical interventions in order to disclose the origin of chronic abdominal pain [24]. However, once central sensitization is established, symptoms can persist in the absence of ongoing abnormal peripheral stimulation. In the present study, only two patients had borne children and none of the patients had a history of abdominal surgery. However, other factors such as viral infection etc. are also well known to be related to nerve injuries; moreover, an important role of genetic factors in the predisposition to develop peripheral neuropathic pain is suggested by animal models, indicating that preexisting factors separate from the degree of neural injury may influence these processes [25]. In this context, our result, i.e. suggestive dysfunction of pelvic nerve may support this pathophysiological hypothesis. In any event, inconsistency of visceral sensitivity between lower and higher pressure of distention might be a key feature to understanding the pathogenesis of FAPS. Paying attention to the fact that FAPS would qualify as a somatoform pain disorder [16], the question we have to ask here is whether there are any pathophysiological relations between FAPS and this disorder with pain localized in other areas. Patients with chronic pain often report abnormal tactile sensitivity in the affected area, suggesting pain disorder may have altered sensory function [26]. But this abnormality was reported to vary from hypoesthenia to allodynia [26]. These lines of evidence suggest that some patients with pain disorder may have a common pathogenesis as FAPS; moreover, this disorder may not be a homogenous but a heterogeneous disease entity. Further study is needed.

Our study has several limitations. It must be noted that there is no proof that this result would be the same for more severe cases. Symptom severity may be related to the sensory profiles assessed in the present study [27]. It is also important to recognize that the report of pain is not the sensation of pain and it is seriously influenced by the decision processes of the individual [28]. Therefore subjects are able to modify the results, even deliberately. As stated in the Rome criteria, ruling out malingering is essential for the diagnosis of FAPS [6] but sometimes it is difficult. Thus, FAPS patients are often suspected to have factitious pain by physicians, and some patients may have a strong desire to prove that they have true pain. It was also reported that patients with FAPS often deny or minimize the role of psychological factors in their abdominal pain [9]. In this context, they may show a pretend attitude such as stoical or sensitive through this examination in order to achieve this desire. These biases are inevitable for this type of experiment, but it would also occur in IBS or even in healthy subjects, suggesting that our results may have some meaning.

## Conclusion

FAPS patients had normal rectal perceptual thresholds, such as discomfort, pain and maximum tolerance and, at the same time, reduced VAS ratings of perceived intensity at the lower pressure of rectal balloon distention. FAPS patients may have hyposensitivity to non-noxious physiological distention with normal sensitivity to painful distention, which may be a key feature to understanding the pathogenesis.

## Competing interests

The authors declare that they have no competing interests.

## Authors' contributions

TN conceptualized and designed the study, collected and analyzed the data, interpreted the results, and drafted the manuscript. MK collected the data. All authors read and approved the final manuscript.
